# Screening and molecular functional analysis of telomere-related genes in abdominal aortic aneurysms based on bioinformatics

**DOI:** 10.3389/fcvm.2026.1716161

**Published:** 2026-03-10

**Authors:** Tao Yuan, Wei Bi, Yang Liu, Huanhuan Sun, Nanqi Cui, Jian Wang, Jiahao Hou, Runbo Song, Xiang Gao

**Affiliations:** 1 Department of Vascular Surgery, Second Hospital of Hebei Medical University, Shijiazhuang, China; 2 Department of General Surgery, Third Hospital of Shijiazhuang City, Shijiazhuang, China

**Keywords:** abdominal aortic aneurysm, differential expression analysis, immune infiltration, machine learning, transcriptional regulatory network

## Abstract

**Background:**

Abdominal aortic aneurysm (AAA) is a life-threatening vascular condition characterized by progressive aortic dilation. However, no effective pharmacotherapies exist to halt its progression. This study aimed to investigate telomere-related molecular signatures in AAA to identify potential targets for therapeutic development.

**Method:**

We obtained the dataset GSE57691, including the RNA-expression data of AAA and normal control samples, from the GEO database. Weighted Gene Co-expression Network Analysis (WGCNA) was performed using the “WGCNA” package to identify AAA phenotype-related gene modules, and differential expression analysis of differentially expressed genes (DEGs) was conducted using the “limma” package. Biomarker identification was achieved through LASSO and Support Vector Machine (SVM) algorithms using the “glmnet” and “e1071” packages. GSEA_4.2.2 was utilized for gene enrichment analysis, and “CIBERSORT” and “estimate” were employed for immune infiltration. An independent single-cell RNA-seq dataset (GSE237230) was analyzed using the Seurat pipeline to characterize the cellular landscape and validate biomarker expression at single-cell resolution. Finally, the hTFtarget and Encori databases were utilized to construct the transcriptional regulatory network, and the “oncoPredict” package was employed to identify potential drugs. We verified the reliability of the model genes *in vitro* via the quantification assays (qPCR, western blot and ELISA) and the phenotypic determination assays (flow cytometry and trans-well).

**Results:**

Following WGCNA, AAA-related modules (MEmagenta/MEpink) were identified. Intersecting gene modules, DEGs, and telomere genes yielded eight candidates, among which KLF15 and ZBTB16 were prioritized via Lasso and SVM. Low expression of these biomarkers correlated with immune-inflammatory activation, whereas high expression was linked to a suppressive microenvironment that alleviated AAA progression. Furthermore, a transcriptional regulatory network was constructed, identifying key target mRNAs and potential drugs. Single-cell analysis confirmed distinct clusters and specific enrichment of ZBTB16 in B cells. Angiotensin II (Ang II) treatment downregulated KLF15 and ZBTB16 in T/G HA-VSMCs. Overexpression of ZBTB16 attenuated Ang II-induced inflammation, apoptosis, and migration and suppressed MMP-2/9 levels, indicating inhibited migratory activity.

**Conclusion:**

We analyzed telomere-related signatures in AAA and identified two crucial biomarkers linked to the immune infiltration of patients. Our findings furthered the understanding of telomere signatures in AAA progression.

## Introduction

Abdominal aortic aneurysm (AAA) is a pathologic and degenerative vascular disease characterized by abdominal aortic dilatation (an aortic diameter >3 cm or greater focal dilation), with an incidence of 4%–8% in developed countries, predominantly affecting men (1, 2). The risk factors of AAA include age > 65 years, previous or current smoking status (the most modiﬁable factor), hypertension, atherosclerosis, and history of aortic dissection or aortic surgery (3). Its pathogenesis is complex and involves multiple biological processes, such as vascular smooth muscle cell apoptosis, oxidative stress, elastin depletion, aortic mural inflammation, and degradation of the extracellular matrix (4). The arterial wall can be remodeled under biochemical, mechanical, and hemodynamic stimuli (5), and its living components must be regenerated to maintain wall integrity and withstand repetitive wall stresses (6). Currently, the standard therapeutic options for patients with AAA diameter ≥5.5 cm are open surgical repair or endovascular exclusion (7). Three potential models support AAA pathogenesis: (Ⅰ) AAA as a purely local disease of atherosclerosis, (Ⅱ) AAA as a systemic dilating disease governed by genotype, and (Ⅲ)AAA as a manifestation of a globally sick or diseased vascular tree caused by a chronic inflammatory process (8). These hypotheses are interconnected; for example, genotype-mediated inflammation can also modify gene expression (9). However, the full range of systemic abdominal aorta with focal manifestation has not been fully categorized, and the development of pharmacological or cell-based therapies to restrict or prevent AAA formation, expansion, and rupture remains an unmet clinical need.

Telomeres are special structures of DNA at the ends of chromosomes with TTAGGG repeats, preventing enzymatic degradation, fusion, and loss or reconstruction of end chromosomes (10–12). With cell division, the “end DNA replication problem” inevitably causes telomere attrition and chromosome instability, but telomerase can add TTAGGG repeats, maintaining telomere integrity (13). Telomere length is closely associated with cell aging, decreasing with cell division number and inﬂuenced by smoking and oxidative stress (14). Vascular tissue undergoes constant hemodynamic and mechanical insults; therefore, a healthy endothelial layer is crucial for the response to repetitive stress. Damaged and dysfunctional endothelial cells need rapid division or are replaced by endothelial progenitor cells and surrounding endothelial cells (15). Evidence suggests that endothelial cells of arteries in high hemodynamic disturbances exhibit increased turnover and telomere shortening (16). For example, the telomere lengths of endothelial cells in the iliac artery were shorter than those from the internal thoracic artery and iliac veins (17). Atturu et al. revealed that shortened telomere length of leukocytes was closely linked to AAA (18). Cafueri et al. demonstrated that smooth muscle and endothelial cells from AAA tissues exhibit increased telomere attrition and oxidative stress (19), suggesting that the telomere signature plays an important role in AAA progression.

In this study, we applied two machine learning methods to screen the potential biomarkers of AAA and explore immune infiltration and pathway activation differences of AAA patients by combining telomere signatures. First, we obtained the RNA-seq data of AAA patients and normal controls from the Gene Expression Omnibus (GEO) database and 2,093 telomere-related genes from the TelNet database. Second, Weighted Gene Co-Expression Network Analysis was performed to identify AAA phenotype-related gene modules, and the differentially expressed genes (DEGs) between AAA and control groups were recognized. Third, Lasso and Support Vector Machines (SVM) algorithms were used to determine two key biomarkers (*KLF15* and *ZBTB16*) from overlapping genes; low levels of these two biomarkers were associated with the activation of immune inflammatory pathways (such as T cell receptor signaling and B cell receptor signaling) and high levels were associated with low infiltration level of naïve B cells, naïve T cells CD4, and memory-activated T cells CD4, suggesting that the high expression of two biomarkers in AAA may inhibit the inflammatory response of vascular tissues and alleviate AAA progression. In addition, we also identified seven targeting miRNA of *KLF15* and 19 targeting miRNA of *ZBTB16* in the transcriptional regulatory network, and several potential targeting drugs of two biomarkers were screened out. Our findings might offer some novel insights into the treatment and intervention strategy of AAA.

## Materials and methods

### Data acquisition

The chip sequencing data of AAA patients (GSE57691) were derived from the Gene Expression Omnibus (GEO) (https://www.ncbi.nlm.nih.gov/geo/) database (20), containing 49 AAA and 10 normal control samples, after removing four outlier samples (21). The telomere-related gene set was provided by the TelNet database (http://www.cancertelsys.org/telnet/), including 2,093 genes.

### WGCNA for trait-related gene modules

The samples were classified into AAA and control groups. Data quality control was first performed using the goodSamplesGenes function to filter out genes with excessive missing values and samples with an overabundance of lowly expressed genes. Weighted Gene Co-expression Network Analysis (WGCNA) was then performed utilizing the “WGCNA” package to screen AAA phenotype-related gene modules (22). The soft-thresholding power was set to 0.90, the minimum module size to 200 genes, and the module merging threshold to 0.2. The modules with the strongest positive or negative correlation of AAA phenotypes were selected as the key modules, and the gene module membership (GM), |module membership (MM)| ≥ 0.8, and gene significance (GS) for correlation coefficient ≥0.4 was used for screening the hub-genes.

### Differentially expressed genes (DEGs) analysis

The differential expression analysis between AAA and control samples was performed by “limma” package. |log_2_ fold change (FC)| ≥ 1 and *p*.adj < 0.05 were set as screening criteria for DEGs (23).

### Lasso and SVM algorithm for potential biomarkers

The overlapping genes among the hub-genes, DEGs, and telomere-related gene set was applied for the gene screening of a prognostic model using two machine learning methods (24). First, least absolute shrinkage and selection operator (Lasso) as a classic shrinkage estimation method was carried out using the “glmnet” package to obtain the potential model. Second, support vector machines (SVM) was applied for the recursive elimination of characteristic genes and performed using the “e1071” package. Finally, the intersection genes between two algorithms were selected as the potential biomarkers (25).

### Gene set enrichment analysis

Based on the median value of biomarker expression, the samples were split into high- and low-expression groups. Next, GSEA, version 4.2.2 was performed to identify significant gene enrichment between the two groups. Kyoto Encyclopedia of Genes and Genomes (KEGG) and Gene Ontology (GO) analyses were conducted using the c2.cp.kegg and c5.go gene set collections, respectively (26). Immune infiltration analysis.

The expression markers of 22 immune-infiltrating cell types (LM22) from the CIBERSORT database (https://cibersortx.stanford.edu/) (27) and the CIBERSORT package were used to calculate the immune infiltration level of 22 immune cells in different samples (24). The rank sum test was used to determine significant differences in infiltration between the tumor and the control groups, and the Spearman method was applied for correlation analysis between the biomarker and immune infiltration cells. Meanwhile, the “estimate” package was also utilized for immune infiltration analysis using the rank sum test, and correlations among the biomarkers and StromalScore, ImmuneScore, and ESTIMATEScore was calculated (28).

### Transcriptional regulatory network analysis of biomarkers

Subsequently, we identified potential transcription factors (TFs) for the biomarkers using the hTFtarget database (https://guolab.wchscu.cn/hTFtarget/). Additionally, biomarker-targeting miRNAs supported by five experimental verifications were analyzed via the ENCORI database (https://rnasysu.com/encori/). The transcriptional regulatory network was then visualized using Cytoscape (version 3.8.0) (29).

### Potential targeted drug analysis

The “oncoPredict” package was used to calculate the inhibitory concentration 50 (IC50) of patients based on drug data from 198 cases from the Genomics of Drug Sensitivity in Cancer2 (GDSC2) database. And the spearman method was employed to identify the drugs with significant correlations with biomarker expression (|cor| ≥0.5 and *p* < 0.01) (30).

### Acquisition and processing of single-cell RNA sequencing (scRNA-seq) data

The scRNA-seq data (31) were obtained from the GEO database (GSE237230). The dataset comprised aortic tissue samples from four patients with AAA, with two samples per patient, resulting in a total of eight samples for analysis. The scRNA-seq data were processed using the Seurat package. Low-quality cells were filtered out based on the following criteria: cells expressing fewer than 200 or more than 6,000 genesand cells with a mitochondrial gene content exceeding 10%. Data normalization was performed using the SCTransform method. As the raw data had already been integrated from four samples into a single Seurat object, no additional batch correction was required. Dimensionality reduction was carried out using the top 50 principal components. A K-nearest neighbor (KNN) graph was constructed based on Euclidean distances using the FindNeighbors function. Cell clustering was then performed with the FindClusters function at a resolution of 0.1. To identify cluster-specific marker genes, differential expression analysis was conducted using the FindAllMarkers function, with thresholds set at logfc.threshold = 0.30, min.pct = 0.25, only.pos = TRUE, and a minimum difference in expression proportion (pct.1 - pct.2) of 0.25. Finally, cell type annotation for the identified clusters was performed by referencing known marker genes in the CellMarker 2.0 database (http://117.50.127.228/CellMarker/).

### Cell culture and treatment

Human thoracic aortic vascular smooth muscle cells (T/G HA-VSMCs, CRL-1999) were acquired from the American Type Culture Collection (ATCC). Cells were incubated in Dulbecco's Modified Eagle Medium (DMEM) with 10% fetal bovine serum (FBS), 100 U/mL penicillin, and 100 μg/ml streptomycin at 37 °C in a humidified atmosphere encompassing 5% CO₂. Experiments were performed utilizing cells from passages 4–5 to ensure phenotypic stability. To model vascular injury and oxidative stress *in vitro*, T/G HA-VSMCs were serum-starved for 12 h prior to treatment and then stimulated with 1 μM angiotensin II (Ang II) for 24 h. This protocol was based on established models of vascular smooth muscle cell (VSMC) activation and dysfunction, which mimic key features of pathological conditions such as hypertension, atherosclerosis, and AAA (32).

### Overexpression and qPCR detection

For ZBTB16 overexpression, the full-length cDNA was cloned into the pLVX-3×FLAG-Puro vector (V013340, Shanghai Newpu Biotechnology, China) and verified by sequencing. Plasmids were transfected into T/G HA-VSMCs using Lipofectamine 3000 (Invitrogen, Waltham, USA). Total RNA was collected from cells (5 × 10^6^) utilizing RNA Isolater Total RNA Extraction Reagent (R401-01, Vazyme, Nanjing, China). After lysis and the addition of chloroform, samples were incubated at 4 °C for 5 min and centrifuged (12,000 × g, 15 min). The aqueous phase was mixed with isopropanol, incubated at 4 °C for 10 min, and centrifuged (12,000 × g, 10 min). RNA pellets were washed with 75% ethanol, briefly air-dried, and dissolved in RNase-free water. RNA concentration was detected by a NanoDrop spectrophotometer, and samples were placed at −80 °C. Genomic DNA was removed with DNase I, and 1 μg of total RNA was reverse-transcribed using oligo(dT) primers and the BeyoRT™ II Kit (D7168S, Beyotime, China) at 42 °C for 1 h, followed by enzyme inactivation at 80 °C for 10 min.

qPCR was performed in 20 μL reactions containing cDNA, gene-specific primers, and SYBR Green Master Mix (D7260, Beyotime) on a real-time PCR system. Cycling conditions were 95 °C for 2 min and then 40 cycles of 95 °C for 15 s and 60 °C for 15 s. Fluorescence was recorded at each cycle. Gene expression was normalized to GAPDH and analyzed through the 2^−ΔΔCt^ method. All experiments included three biological and technical replicates. Primer sequences are displayed in Table 1.

**Table 1:** Specific primer for qPCR.

Gene	Forward (5′-3′)	Reverse (5′-3′)
KLF15	GTGAGAAGCCCTTCGCCTGCA	ACAGGACACTGGTACGGCTTCA
ZBTB16	GAGCTTCCTGATAACGAGGCTG	AGCCGCAAACTATCCAGGAACC
GAPDH	GTCTCCTCTGACTTCAACAGCG	ACCACCCTGTTGCTGTAGCCAA

### ELISA analysis

The concentrations of pro-inflammatory cytokines IL-1β (CB10347-Hu), IL-6 (CB10373-Hu), and TNF-α (CB11762-Hu) in cell culture supernatants were determined utilizing commercially available human ELISA kits, including IL-1β (CB10347-Hu), IL-6 (CB10373-Hu), and TNF-α (CB11762-Hu) from the Coibo biotechnology company (Shanghai, China). 50 μL of standard or sample was added to pre-coated wells and placed at 37 °C for 1 h. After three washes, 50 μL of biotin-conjugated detection antibody was added to each well and stored at 37 °C for 1 h. Then, 50 μL of termination solution was added, and absorbance was tested at 450 nm employing a microplate reader.

### Flow cytometry analysis of apoptosis

The impact of *ZBTB16* overexpression on Ang II-induced apoptosis in T/G HA-VSMCs was measured via flow cytometry utilizing the Annexin V-FITC Apoptosis Detection Kit (C1062S, Beyotime, China). oe-NC or oe-ZBTB16-transfected cells were treated with 1 μm Ang II for 24 h, harvested by centrifugation (1,000 *g*, 5 min), washed with cold PBS, and resuspended in binding buffer. Annexin V-FITC (5 μL) and PI (10 μL) were added, and cells were incubated in the dark at 25 °C for 10 min. Samples were kept on ice and analyzed immediately. Apoptotic populations were quantified based on Annexin V and PI staining (FL1/FL3), with compensation set using unstained and single-stained controls.

### Trans-well assay for cell migration

Cell migration was evaluated using BeyoGold™ Transwell inserts (FTW010-6Ins, 8 μm pore; Beyotime, Shanghai, China). oe-NC or oe-ZBTB16-transfected T/G HA-VSMCs were serum-starved for 12 h and seeded (5 × 10^4^ cells) into the upper chamber in 300 μL of serum-free DMEM. The lower chamber contained 700 μL of DMEM with 10% FBS as chemoattractant. Cells were treated with 1 μm Ang II for 24 h to induce invasive behavior. Non-invading cells on the upper surface were removed with a cotton swab, and invaded cells on the lower membrane were fixed with 4% paraformaldehyde, stained with 0.5% crystal violet, and imaged under a light microscope (Eclipse Ti2-A, Nikon). Cells in three random fields were counted for quantification. Experiments were carried out in triplicate and repeated independently three times (33).

### Western blot

Proteins were extracted from T/G HA-VSMCs using ice-cold RIPA buffer containing protease and phosphatase inhibitors (R0010, Solarbio, Beijing, China), and concentrations were measured by BCA assay. Equal amounts of protein (30 μg per lane) were separated by 10% SDS-PAGE and transferred to PVDF membranes. Membranes were blocked with 5% skim milk in TBST for 1 h and incubated overnight at 4 °C with primary antibodies against MMP-2 (ab92536, 1:1000, Abcam), MMP-9 (ab76003, 1:1000, Abcam), and β-actin (ab8227, 1:1000, Abcam). After washing, membranes were incubated with HRP-conjugated goat anti-rabbit IgG (ab6721, 1:2000) for 1 h at room temperature. Proteins were visualized employing ECL and quantified by ImageJ software.

### Statistical analysis

All statistical tests were conducted using the R software (version 4.0.5) and Sangerbox 2 (34). Data from *in vitro* experiments were analyzed using GraphPad Prism software (version 10.6) and were presented as mean ± standard deviation (SD). The normality of continuous variables was assessed using the Shapiro–Wilk test. For comparisons between two independent groups, the two-tailed Student's *t*-test was applied for data following a normal distribution; otherwise, the nonparametric Wilcoxon rank-sum test (also known as the Mann–Whitney *U*-test) was used. Correlations between two continuous variables were evaluated using Spearman's rank-order correlation. A *p*-value <0.05 was considered statistically significant.

## Results

### WGCNA for the AAA phenotype-related model genes

**Figure 1:**
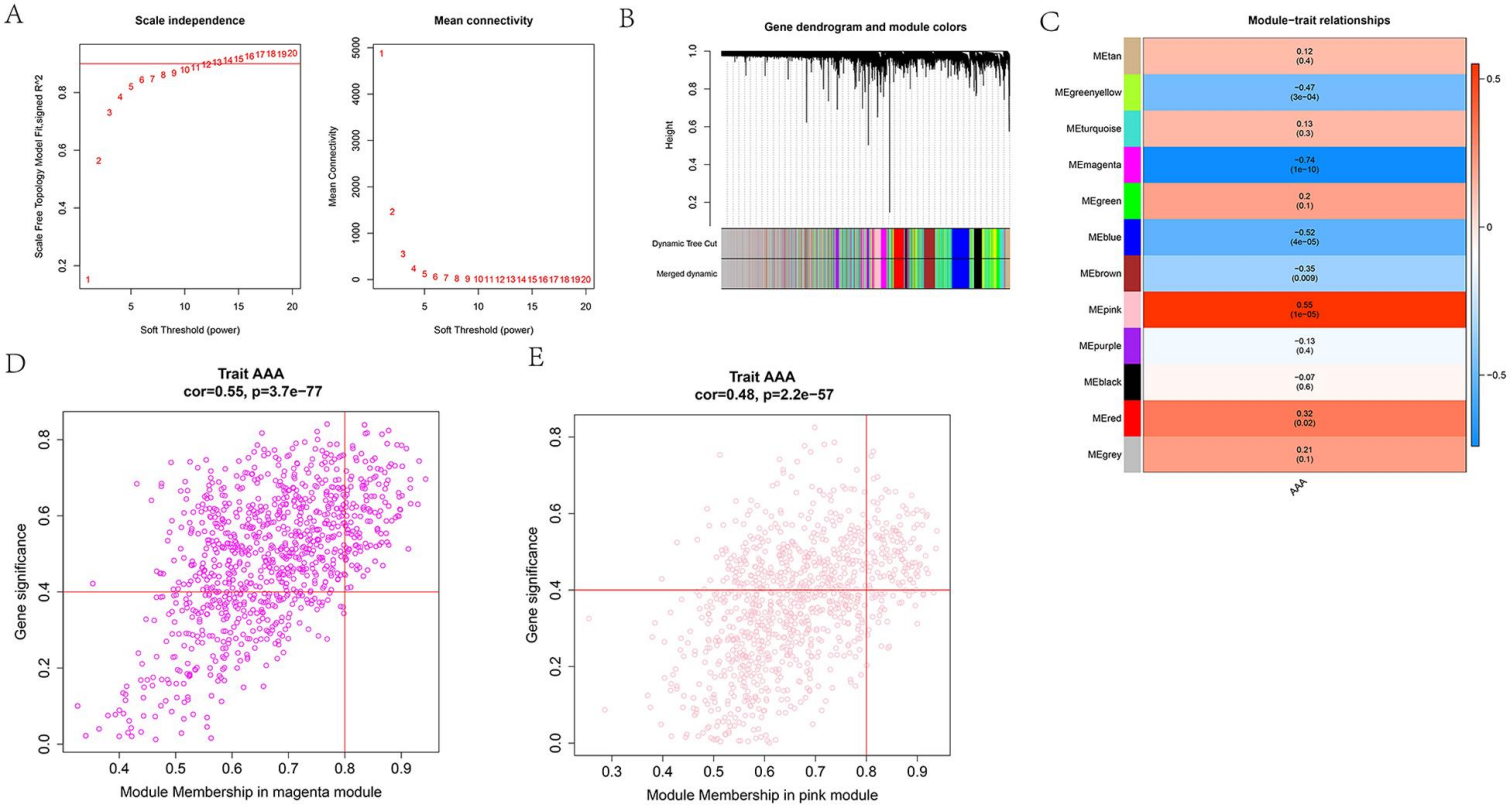
WGCNA for AAA-related gene modules. **(A)** Soft threshold screening plot. **(B)** Gene modules and merging. **(C)** The correlation analysis between module and abdominal aortic aneurysm (AAA)-phenotype (red denotes the positive correlation and blue denotes the negative correlation). **(D)** Correlation analysis of magenta genes and AAA-phenotype. **(E)** Correlation analysis of pink genes and AAA-phenotype.

We performed the WGCNA in the GSE57691 database and selected the soft threshold of 13 for topology network construction (Figure 1A). After setting the minimum number of module genes and correlation merging, we obtained 12 modules, in which the grey module is an invalid module (Figure 1B). The correlation analysis revealed that the MEmagenta module was markedly negatively correlated with the AAA phenotype (cor = −0.74 and *p* = 1 × 10^−10^) and the MEpink module was notably positively associated with the AAA phenotype (cor = 0.55 and *p* = 1 × 10^−5^) (Figure 1C). Next, we screened 136 hub-genes from the magenta module and 113 hub-genes from the pink module with the criteria of GS ≥0.4 and |MM| ≥0.8 (Figures 1D,E).

### Identifying KLF15 and ZBTB16 as potential biomarkers

**Figure 2:**
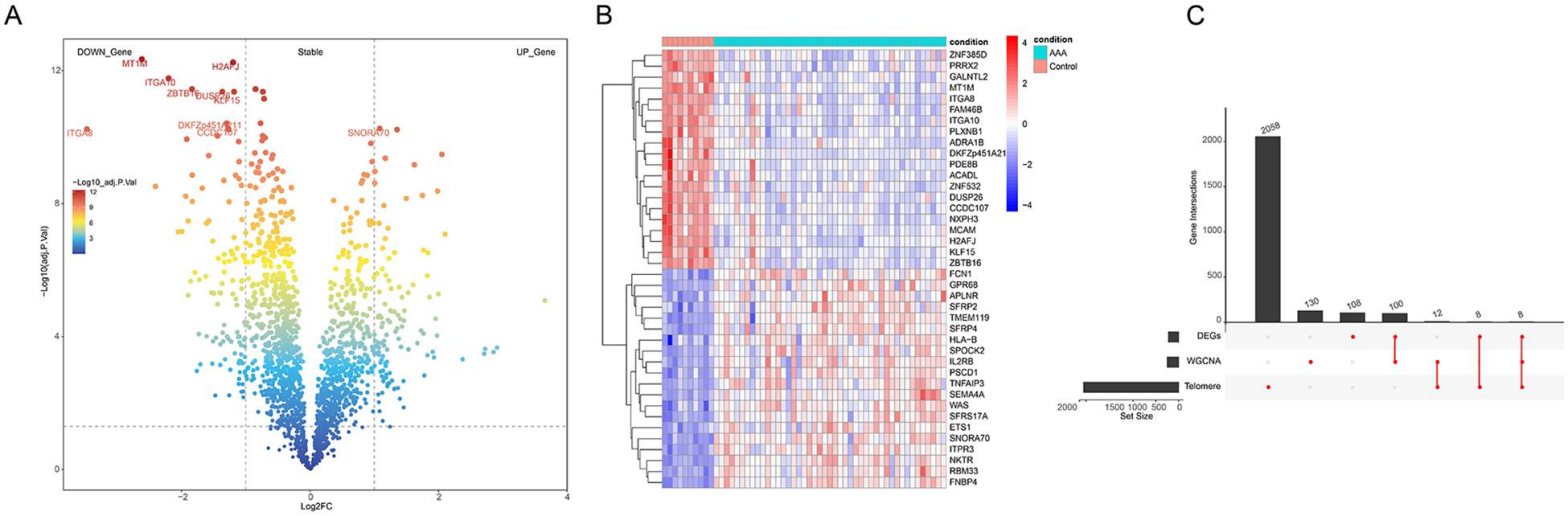
Differential expression analysis. **(A)** Volcano plot of differentially expressed genes (DEGs) between tumor and control groups (the left are down-regulated genes and the right are up-regulated genes). **(B)** The heatmap of the top20 DEGs (the blue denotes low-expression and the red denotes high-expression). **(C)** The UpSet plot of overlapping genes among DEGs, hub-genes, and telomere-related genes.

**Figure 3:**
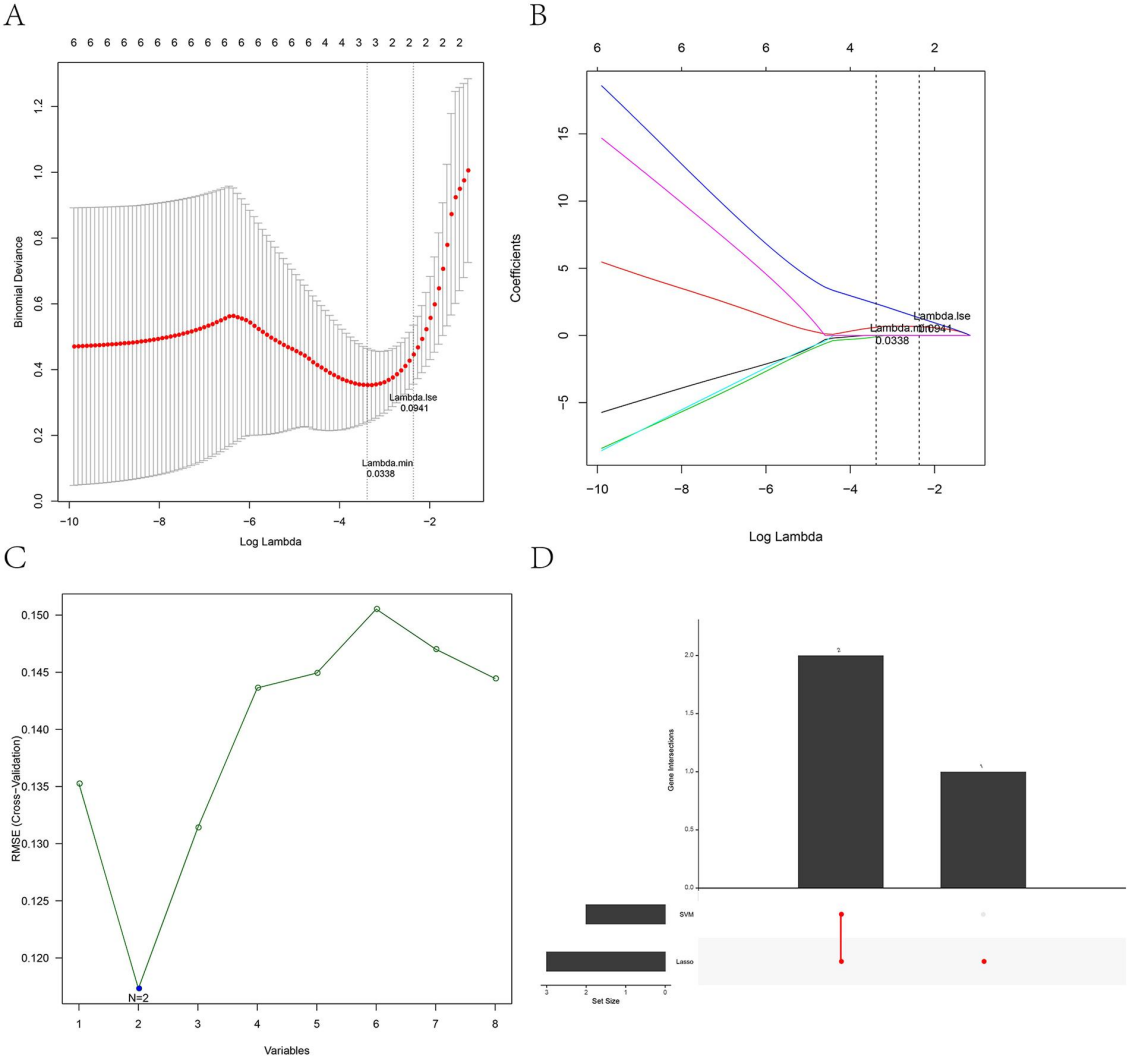
Identifying the key biomarkers. **(A)** Lasso penalty term parameter diagram (The dotted line on the left is where the cross-validation error is minimal). **(B)** Lasso regression coefficient diagram [the abscissa is log(lambda) and the ordinate is the coefficient of the gene]. **(C)** The relationship between SVM-RFE universal error and feature number (the horizontal coordinate is the number of genes included in each iteration model, and the vertical coordinate is the root-mean-square error). **(D)** The intersection UpSet maps of characteristic genes were obtained by Lasso and SVM-RFE.

We screened the DEGs between AAA and control samples with |log2FC| ≥1 and p.adj <0.05 criterion and obtained 224 DEGs, namely 111 up-regulated DEGs and 113 down-regulated DEGs (Figure 2A); the expression heatmap of the top 20 up- and down-DEGs is shown in (Figure 2B). The intersection among the hub-genes, DEGs, and telomere-related genes produced eight AAA-related differentially expressed telomere genes (Figure 2C). Further, we performed the Lasso cox regression analysis on eight candidate genes and selected the lambda.min = 0.0338 as model result: a total of three feature genes, *KLF15*, *HCLS1*, and *ZBTB16*, were obtained (Figures 3A,B). In addition, the SVM analysis of eight candidate genes revealed that the model has the minimum error when *N* = 2 and included two feature genes, *KLF15* and *ZBTB16* (Figure 3C). The intersection between the lasso and SVM results generated two key genes (*KLF15* and *ZBTB16*) as potential biomarkers (Figure 3D).

### Low levels of two biomarkers were associated with immune inflammatory activation

**Figure 4:**
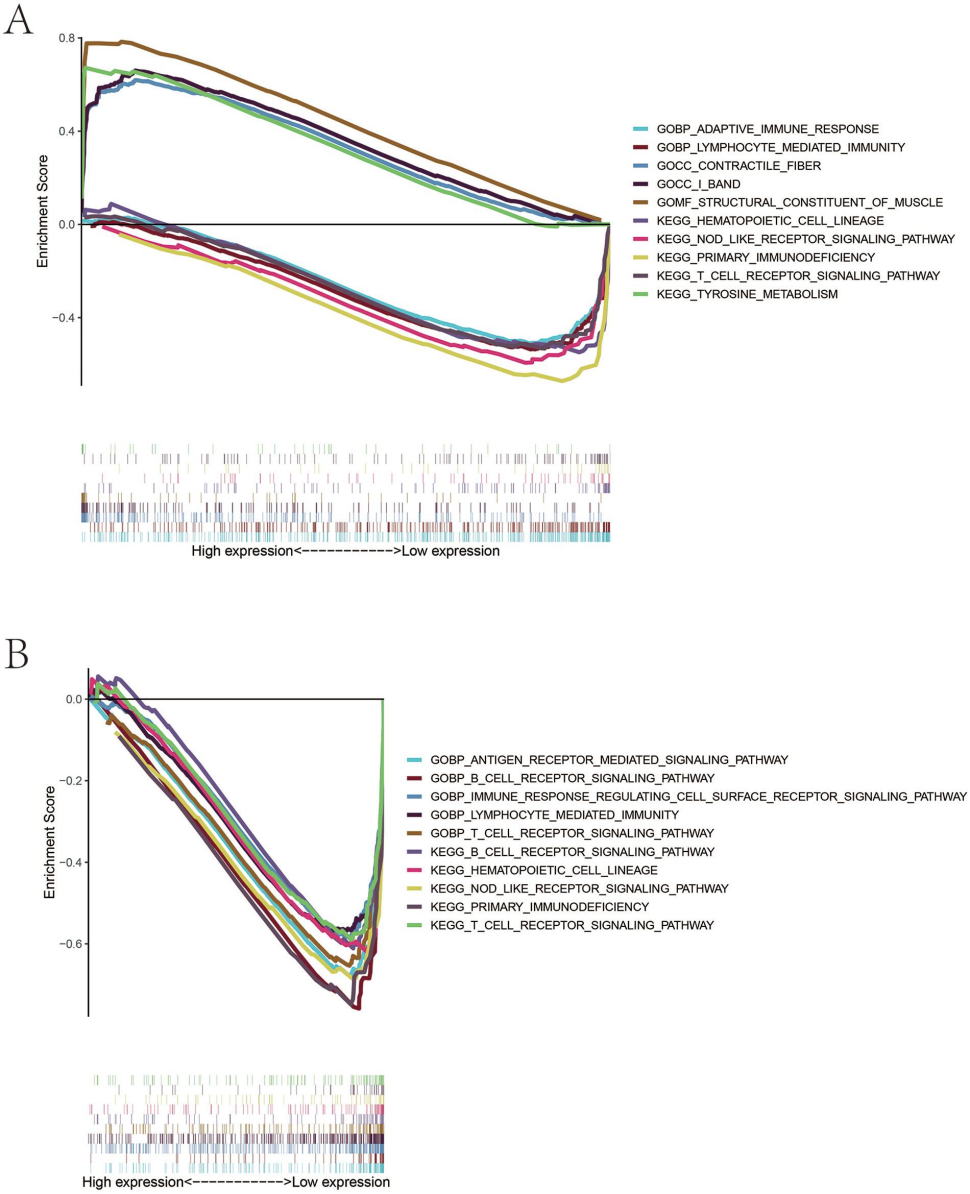
Gene Set enrichment analysis (GSEA) reveals the biological functions of KLF15 and ZBTB16 expression in the development of AAA. **(A)** GSEA plots for the high- and low-expression groups of *KLF15*. Pathways significantly enriched in the *KLF15* low-expression group include primary immunodeficiency, NOD-like receptor signaling, adaptive immune response, and hematopoietic cell lineage. Representative enrichment plots are shown. **(B)** GSEA plots for the high- and low-expression groups of *ZBTB16*. Pathways significantly enriched in the *ZBTB16* low-expression group include antigen receptor-mediated signaling, T cell receptor signaling, B cell receptor signaling, hematopoietic cell lineage, primary immunodeficiency, and NOD-like receptor signaling. Representative enrichment plots are shown.

We split the patients into high- and low-biomarker expression groups based on *KLF15* and *ZBTB16* expression. The GSEA_4.2.2 software was used to perform the gene set enrichment analysis and it was shown that patients with high expression of *KLF15* were associated with tyrosine metabolism, contractile fiber, and the structural constituent of muscle pathways, while low levels of *KLF15* were associated primary immunodeficiency, NOD-like receptor signaling, adaptive immune response, and hematopoietic cell lineage pathway activation (Figure 4A). For *ZBTB16*, its low expression was associated with antigen receptor-mediated signaling, T cell receptor signaling, B cell receptor signaling, hematopoietic cell lineage, primary immunodeficiency, and NOD-like receptor signaling pathway activation (Figure 4B).

### Biomarkers of KLF15 and ZBTB16 affected immune infiltration

**Figure 5:**
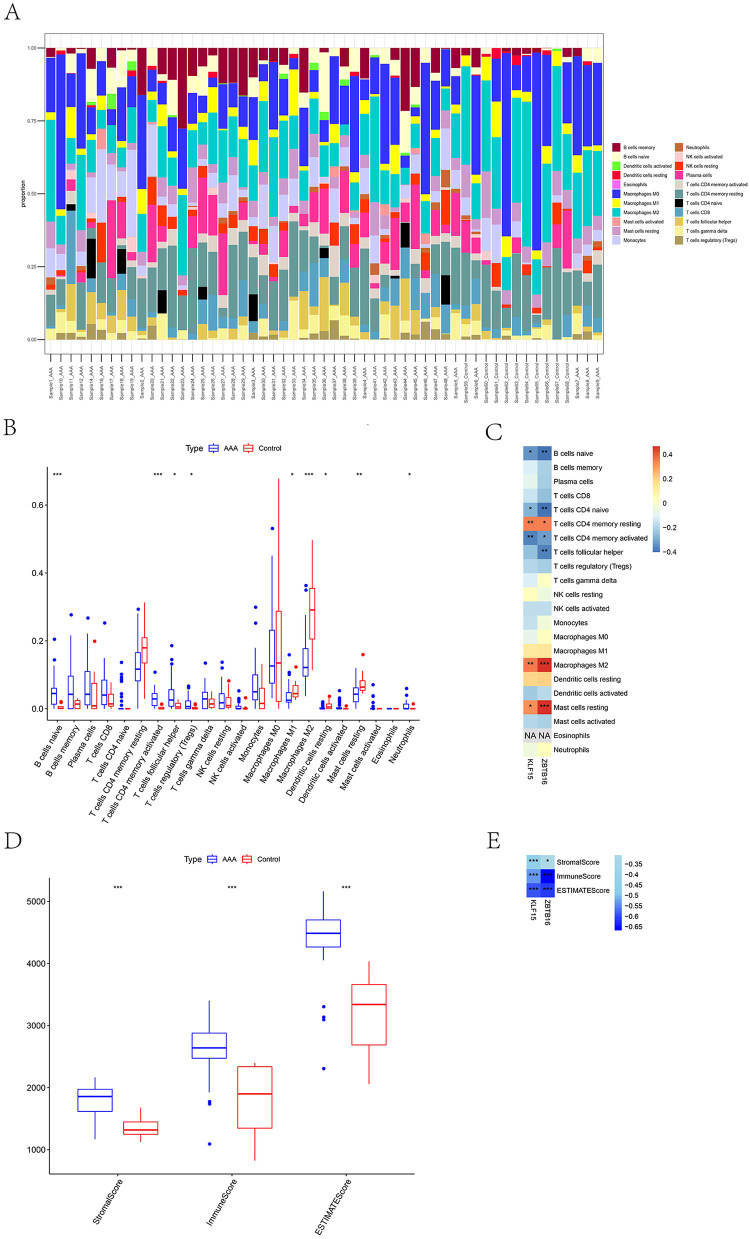
Immune infiltration landscape and its association with KLF15 and ZBTB16 expression in AAA. **(A)** Stacked bar plot generated by CIBERSORT analysis, depicting the relative proportions of 22 immune cell types across all individual samples from the AAA and control groups. **(B)** Box plots comparing the infiltration levels of 22 immune cell types between the AAA group and the control group. **(C)** Heatmap of Spearman correlation coefficients between the expression levels of the two biomarkers (*KLF15* and *ZBTB16*) and the infiltration levels of significantly different immune cell types. **(E)** Spearman correlation between the expression levels of *KLF15* and *ZBTB16* and the ESTIMATE score. ns, not significant, *p < 0.05, **p < 0.01, ***p < 0.001.

CIBERSORT analysis of immune infiltration revealed significant differences in nine immune cell types between the AAA and control groups (Figure 5A). Specifically, naïve B cells, activated CD4 memory T cells, follicular helper T cells, regulatory T cells (Tregs), and neutrophils were significantly increased in the AAA group. In contrast, M1 macrophages, M2 macrophages, resting dendritic cells, and resting mast cells were markedly increased in the control group (Figure 5B). Correlation analysis displayed that the memory-resting CD4 T cells, macrophage M2, and resting mast cells were significantly positively correlated with two biomarkers, whereas the naïve B cells, naïve CD4 T cells, and memory-activated CD4 T cells were notably negative correlated with two biomarkers (Figure 5C). In addition, the stroma score, immune score, and ESTIMATE score in the AAA group were markedly higher than that in control group (Figure 5D) and were negatively related to biomarker expression (Figure 5E). These outcomes suggest that the high levels of the two biomarkers may indicate a suppressive immune microenvironment that decreases the inflammatory response of vascular tissues and alleviates AAA progression.

### Potential transcriptional regulatory targets and drugs of two biomarkers

**Figure 6:**
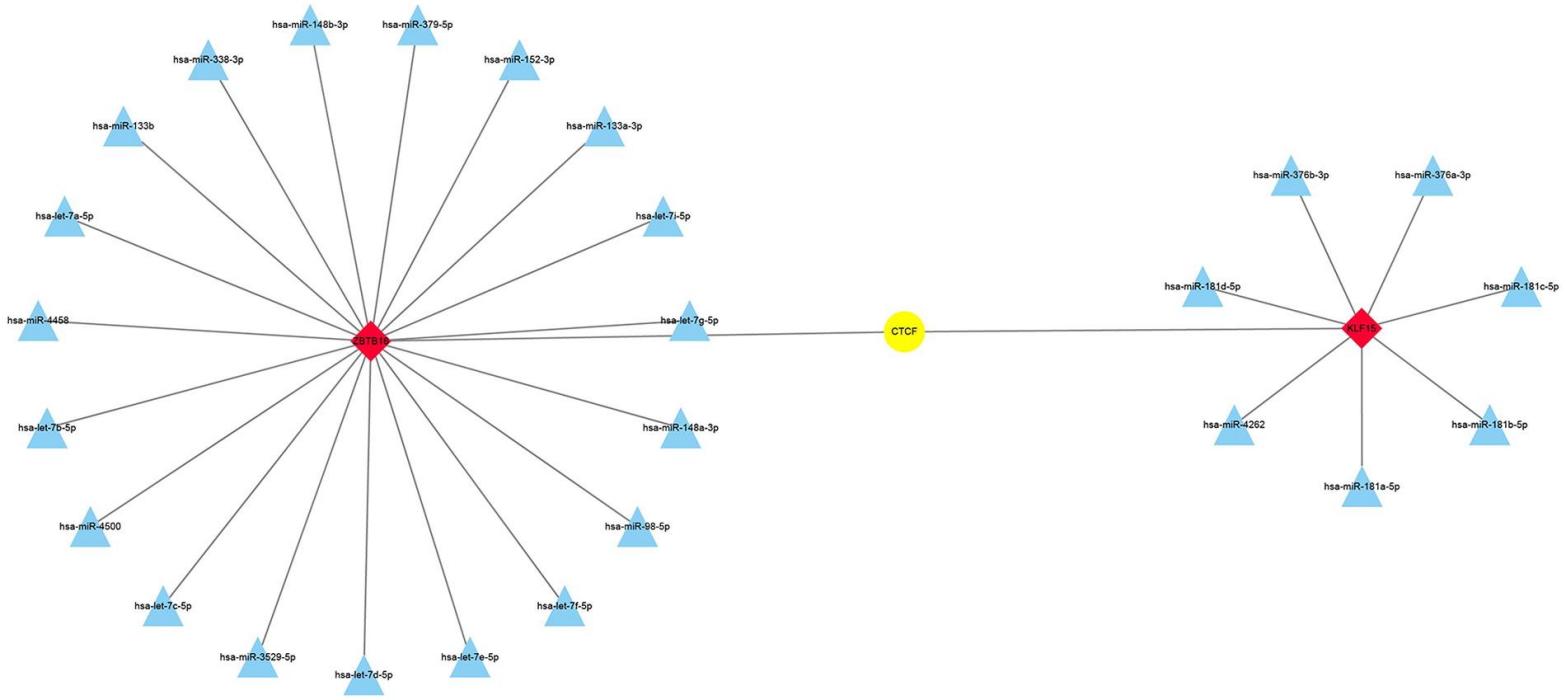
TF regulatory network plot (red represents biomarkers, yellow represents TF transcription factors, and blue represents miRNAs).

**Figure 7:**
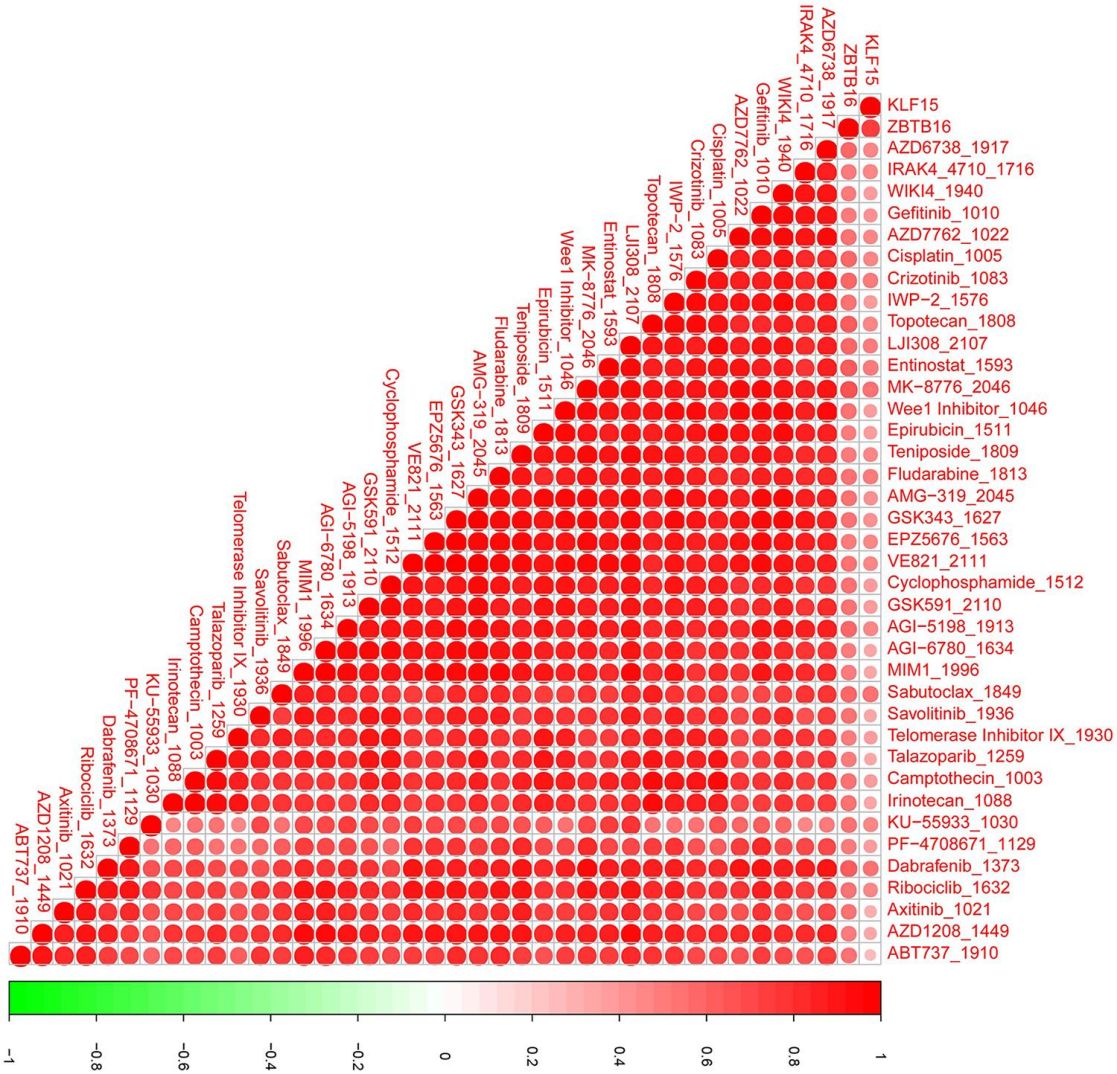
Correlation between candidate drug sensitivity and the expression of KLF15 and ZBTB16 in AAA samples. This heatmap illustrates the Spearman correlation coefficients between the expression levels of the two key biomarkers (*KLF15* and *ZBTB16*) and the predicted half-maximal inhibitory concentration (IC_50_) values for a panel of candidate drugs.

We further screened for the TFs of the two biomarkers using the hTFtarget database, identifying CTCF as a common TF. Additionally, the ENCORI database identified 7 miRNAs targeting KLF15 and 19 targeting ZBTB16. The TF regulatory network was visualized using Cytoscape (Figure 6). Drug prediction analysis showed that four drugs, namely Crizotinib_1083, Mk-8776_2046, Sabutoclax_1849, and Dabrafenib_1632, were positively correlated with *KLF15* expression, while Topotecan_1808, LJI308_2017, Entinostat_1593, and MK-8776_2046 were correlated with *ZBTB16* expression (Figure 7).

### scRNA-Seq atlas of AAA

To further validate and elucidate the cellular context of the identified telomere-related signatures in AAA pathogenesis, we performed scRNA-seq analysis on dissociated cells from AAA samples. Following data preprocessing steps including cell filtering, normalization, dimensionality reduction, and clustering, we identified nine distinct cell clusters (Supplementary Figures S1A,B). The cellular composition across samples was visualized using a stacked bar plot (Supplementary Figure S1C). Specific marker genes were used to define major populations: T cells (*CD3E*, *CD3D*, *TRBC1*, and *TRBC2*); macrophages (*LYZ*, *C1QA*, *C1QB*, and *C1QC*); endothelial cells (*VWF*, *PLVAP*, *PECAM1*, *CAV1*); B cells (*CD79A*, *MS4A1*, *CD37*, and *LY9*); fibroblasts (*COL1A2*, *DCN*, *LUM*, and *VCAN*); proliferating leukocytes (*STMN1*, *CORO1A*, and *CD52*); vascular smooth muscle cells (VSMCs) (*ACTA2*, *MYH11*, *TAGLN*, and *RGS5*); neutrophils (*S100A8*, *S100A9*, *CSF3R*, and *CXCL8*); and plasma cells (*IGHG1*, *IGHG2*, *IGHG3*, and *IGHG4*). The expression patterns of these definitive markers are presented in a bubble plot and a heatmap (Supplementary Figures S1D,E). Furthermore, *ZBTB16* showed significantly elevated expression specifically within the B cell cluster (Supplementary Figure S1F). This pattern suggests that high expression of this telomere-related genes may represent a molecular feature of B cell activation, proliferation, or stress adaptation, potentially indicating its close involvement in the immune response associated with AAA.

### ZBTB16 protects against Ang II-induced VSMC dysfunction

**Figure 8:**
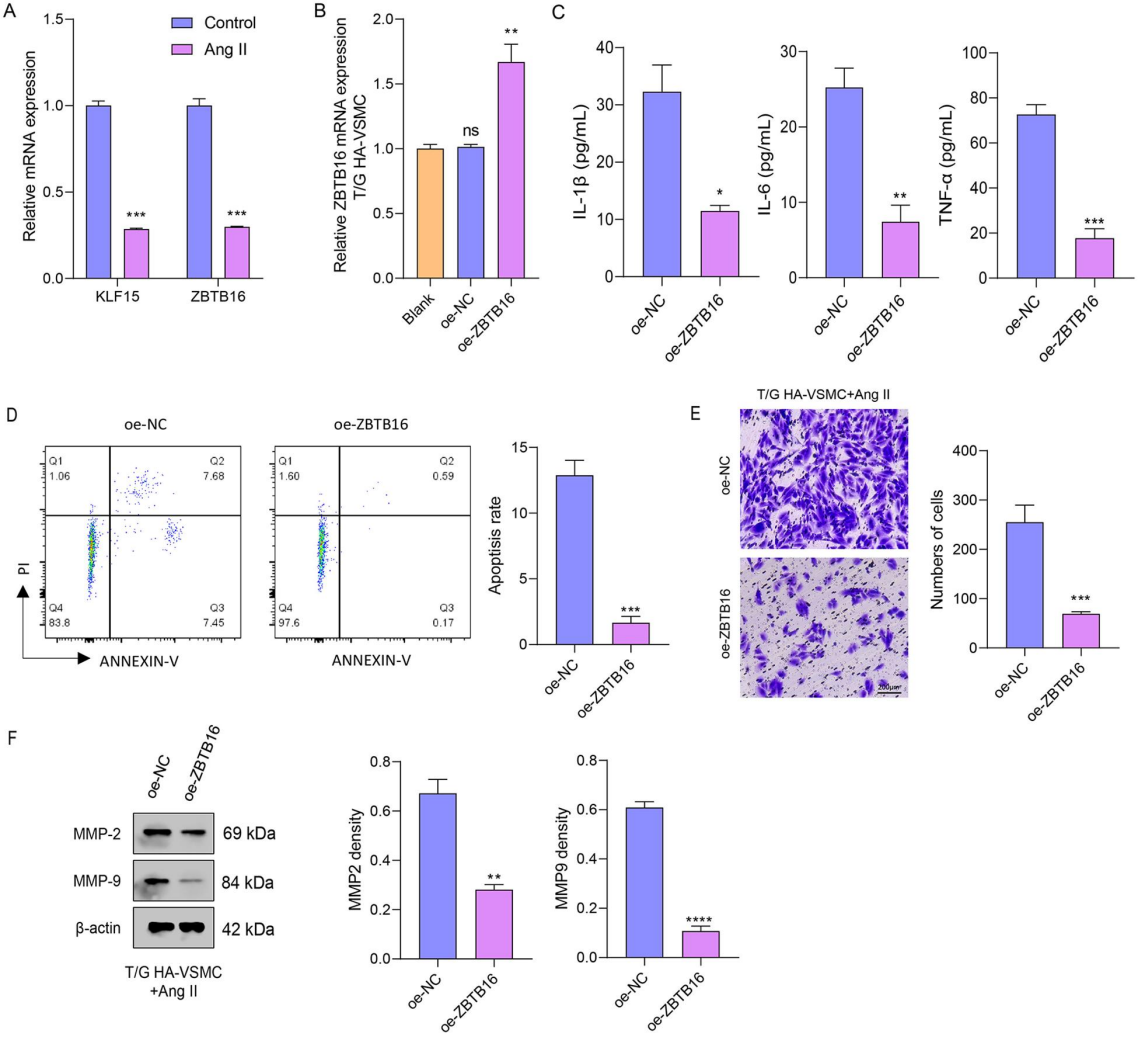
ZBTB16 ameliorates Ang II-induced inflammatory response, apoptosis, and migration in T/G HA-VSMCs. **(A)** qPCR for key gene (*KLF15* and *ZBTB16*) expression after Ang Ⅱ treatment and the overexpression efficiency detection. **(B)** qPCR validation of ZBTB16 overexpression efficiency in T/G HA-VSMCs transfected with oe-*ZBTB16* compared to blank and oe-NC controls. **(C)** Enzyme-linked immunosorbent assay (ELISA) measurement of pro-inflammatory cytokine (IL-1β, IL-6, and TNF-α) concentrations in the supernatants of oe-NC and oe-*ZBTB16* cells treated with Ang II. **(D)** Flow cytometric analysis of apoptosis using Annexin V/PI staining. **(E)** Trans-well test for cell migration. **(F)** Western blot analysis of matrix metalloproteinase MMP-2 and MMP-9 protein levels in oe-NC and oe-ZBTB16 cells following Ang II treatment. β-actin served as the loading control. (*p < 0.05, **p < 0.01, ***p < 0.001, ****p < 0.0001, and ns indicates no significant difference).

To assess Ang II-induced injury, cells were treated with 1 μM Ang II for 24 h. mRNA levels of *KLF15* and *ZBTB16* were quantified by qPCR in control, Ang II-treated, and *ZBTB16*-overexpressing cells. qPCR analysis displayed that the mRNA expression of *KLF15* and *ZBTB16* was markedly more reduced in T/G HA-VSMCs following Ang II treatment compared to the control group (Figure 8A). Additionally, qPCR analysis confirmed efficient *ZBTB16* overexpression in T/G HA-VSMCs transfected with oe-*ZBTB16*, showing markedly higher mRNA levels compared to blank and oe-NC controls (Figure 8B). *ZBTB16* overexpression markedly reduced IL-1β, IL-6, and TNF-α secretion in Ang II-treated T/G HA-VSMCs compared to oe-NC (Figure 8C), indicating that *ZBTB16* attenuates Ang II-induced inflammation. Meanwhile, *ZBTB16* overexpression significantly reduced apoptosis in Ang II-treated T/G HA-VSMCs after 48 h compared to the oe-NC group (Figure 8D), indicating its anti-apoptotic role. *ZBTB16* overexpression inhibited migration in Ang II-treated T/G HA-VSMCs, with significantly fewer migrated cells observed at 48 h compared to oe-NC (Figure 8E). Correspondingly, *ZBTB16* reduced MMP-2 and MMP-9 protein levels (Figure 8F), indicating that it suppresses cell migration by downregulating key matrix metalloproteinases.

## Discussion

AAA is a permanent, pathological dilation of the abdominal aorta characterized by a thinning of the aortic wall and an increased susceptibility to rupture. Several risk factors of AAA, such as genes, age, and smoking, were determined, but its exact pathogenesis is complex and unknown. Despite the effectiveness of open repair surgery and endovascular aortic repair (EVAR), the limitations of poorer long-term durability, of the limited applicability to women with small-diameter vasculature, and of potential lifelong surveillance with this technique are inevitable. Thus, studies to address these issues and develop effective therapeutic agents that prevent AAA growth are required. In this study, we combined telomere signatures and discovered two biomarkers for AAA patients; these biomarkers were associated with the immune pathway activation and immune cell infiltration that affected AAA progression. In addition, the potential targets of two biomarkers were also established; these could be utilized in treatment options. Our study is significant in its aim to understand the pathogenesis of AAA and achieve effective targeted drug selection.

Krüppel-like factor 15 (KLF15) is a zinc-finger transcription factor that inhibits the activation of NF-κB signaling, thereby suppressing tissue inflammation (35). And the overexpression of *KLF15* is crucial for mitigating MAPK and NF-κB pathway-mediated inflammation from injury or post-myocardial infarction (36). Song et al., revealed that high expression of *KLF15* prevented atherosclerosis progression (37), indicating that *KLF15* plays an important role in decreasing vascular inflammation and maintaining vascular health. Our single-cell transcriptomic analysis revealed a specific enrichment of *ZBTB16* expression within the B-cell compartment of AAA tissue. This finding suggests that high *ZBTB16* expression may not be a general feature but is potentially linked to a distinct functional state of these immune cells. *ZBTB16* is recognized as a transcriptional regulator, with established roles in the development and function of innate lymphoid cells (ILCs) and certain T-cell subsets, such as NKT cells (38, 39). However, its expression pattern and functional significance in B cells remain less clearly defined. Our data indicate that, within the AAA microenvironment, elevated *ZBTB16* expression is specifically associated with B cells, possibly marking a unique functional state for this lineage. Existing literature supports a significant role for B cells and antibodies in driving the intramural inflammation and immune responses characteristic of AAA, for instance through antigen presentation, production of pro-inflammatory cytokines, or autoantibodies that exacerbate vascular wall damage (40, 41). Therefore, we propose that *ZBTB16* might participate in regulating adaptive immune responses in AAA by modulating B-cell processes such as activation, proliferation, or antibody class-switching. Its high expression could reflect an adaptive or activated state of B cells under the sustained pressure of chronic vascular injury and inflammation. Future studies employing functional experiments—for example, conditional knockout or overexpression of *ZBTB16* specifically in B cells, combined with *in vivo* AAA models—are warranted to directly elucidate its precise mechanistic role in modulating B-cell function and influencing AAA progression.

The development of AAA is typically characterized by the gradual formation of a mural thrombus (42). These thrombi frequently contain high concentrations of proteolytic enzymes, pro-inflammatory cytokines, and a variety of immune cells, including macrophages, neutrophils, lymphocytes, mast cells, and natural killer (NK) cells (43). Furthermore, the sequestration of circulating cells, platelets, fibrinogen, and lipoproteins from the blood mediates inflammatory responses, increases vessel wall stress, and impairs nutrient delivery, ultimately promoting AAA expansion (44). In the rat AAA model, platelet inhibition was shown to limit AAA development (45), and β-receptor blockers, angiotensin-converting enzyme inhibitors (ACEI), and non-steroidal anti-inflammatory drugs were used for conservative AAA therapy (46). Correlation analysis demonstrated that memory-resting CD4 T cells , macrophage M2 cells, and resting mast cells were markedly positively correlated with two biomarkers, whereas naïve B cells, naïve CD4 T cells, and memory-activated CD4 T cells were notably negatively correlated with two biomarkers. Forester et al., revealed that the T-cells released pro-inflammatory factors and provided B-cells with activated signals for tissue inflammation in AAA (47). The potentiation of inflammation mediated by NK cells is another feature of AAA (48). The high levels of two biomarkers may indicate the high infiltration of immunosuppressive cells (macrophage M2 and resting cells) and lower amount of immune-activated cells (T and B cells), demonstrating these biomarkers are two protective factors of AAA and act to decrease the inflammatory response. CCCTC-binding factor (CTCF) is a shared zinc finger (ZF) transcription factor (49) of *KLF15* and *ZBTB16*; *CTCF* regulates the cell identity genes transcription and determines the cell identity (50). CTCF as a Tcf1 cofactor regulates the cytokine markers of IL-7 and IL-15, promoting the homeostatic proliferation of CD8T cells (51), thus CTCF may also interact with *KLF15* and *ZBTB16*, regulating immune cell proliferation in AAA progression. In addition, we also identified several potential targeted drugs of two biomarkers, namely MK-8776_2046 and sabutoclax_1849, which may provide some reference for AAA treatment.

While this study provides novel insights into the role of telomere-related signatures in AAA pathogenesis, several limitations should be acknowledged. These constraints also highlight clear directions for future investigation. First, the bioinformatic discovery phase relied primarily on a single bulk RNA-seq dataset. Although we validated key findings using an independent single-cell RNA-seq dataset, which offers superior cellular resolution, the prognostic or diagnostic generalizability of the identified biomarkers requires confirmation in larger, independent cohorts with diverse demographics and clinical outcomes. Future work should prioritize assembling a multi-center, multi-platform transcriptomic repository for AAA to rigorously test the robustness of these and other candidate markers. Second, the functional characterization remained incomplete. *ZBTB16* was validated *in vitro* (Ang II-treated VSMCs), but parallel experiments for *KLF15* were not conducted, leaving its specific role in AAA less substantiated. Furthermore, the *in vitro* model captures only certain aspects of AAA pathology. Subsequent research should include functional studies on *KLF15* using gain- and loss-of-function approaches in relevant cell types. More importantly, the definitive role of both genes, particularly the B-cell-enriched *ZBTB16*, must be tested *in vivo* using cell-specific knockout or transgenic mouse models crossed with established AAA models. Third, the predicted drug associations, while analytically significant, are derived from a cancer cell line database. Their direct relevance to AAA pathophysiology is speculative. A more targeted approach would involve high-throughput screening of compound libraries in primary human AAA-derived cells or in *ex vivo* aortic culture systems to identify modulators of *KLF15*/*ZBTB16* pathways with genuine therapeutic potential. Fourth, our single-cell analysis, though informative, was limited to a relatively small sample size from a single cohort. Deeper sequencing and increased sample numbers are needed to fully resolve rare but potentially important cellular subpopulations and their interactions. Finally, this study focused on tissue-intrinsic mechanisms. Whether the dysregulation of these telomere-related genes is reflected in accessible biofluids (e.g., plasma or peripheral blood mononuclear cells) and whether it is specific to AAA compared to other aortic pathologies remain open and clinically pertinent questions. Future efforts should integrate proteomic or circulating RNA analysis of patient blood samples to explore the translational utility of these findings.

## Conclusion

We explored the telomere signature in AAA and developed two key biomarkers (*KLF15* and *ZBTB16*) for AAA patients through the lasso and SVM algorithms. These two biomarkers were relevant to immune cell infiltration and affected immune pathway activation. *CTCF* as a co-factor controlled immunity homeostasis. Our study is expected to offer novel insights into AAA treatment.

## Data Availability

The datasets generated and/or analyzed during the current study are available in the [GSE57691] repository, [https://www.ncbi.nlm.nih.gov/geo/query/acc.cgi?acc=GSE57691] and is also available at Jianguoyun link: https://www.jianguoyun.com/p/DcaS5m0QkufeDRiP24kGIAA.
