# Supplementary figures and images for: Screening and molecular functional analysis of telomere-related genes in abdominal aortic aneurysms based on bioinformatics
Source: Front Cardiovasc Med. 2026 Mar 10;13:1716161. doi: 10.3389/fcvm.2026.1716161 (PMC13008704; doi:10.3389/fcvm.2026.1716161)

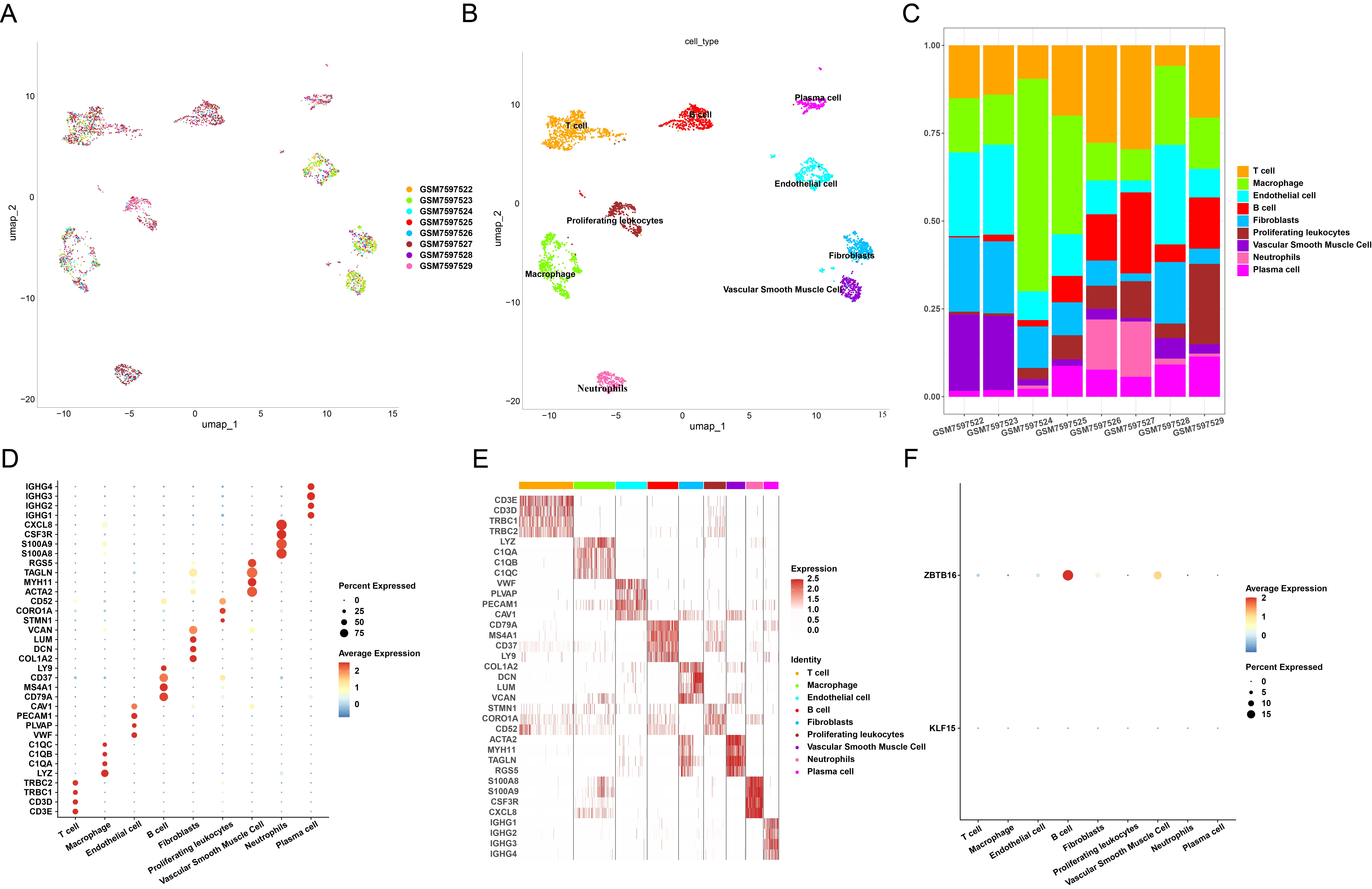

Supplement: Supplementary file 1 [file Image1.jpeg]
